# Coexistence of atypical adenomatous hyperplasia, minimally invasive adenocarcinoma and invasive adenocarcinoma: Gene mutation analysis

**DOI:** 10.1111/1759-7714.13798

**Published:** 2021-01-14

**Authors:** Junya Song, Yinghui Xu, Zhiguang Yang, Yunpeng Liu, Peng Zhang, Xu Wang, Chao Sun, Ye Guo, Shi Qiu, Guoguang Shao, Kewei Ma

**Affiliations:** ^1^ Cancer Center The First Hospital of Jilin University Changchun China; ^2^ Thoracic Surgery Department The First Hospital of Jilin University Changchun China

**Keywords:** Multiple primary lung cancer (MPLC), mutation spectrum, phylogenetic tree, tumor mutation burden (TMB), whole exome sequencing (WES)

## Abstract

Multiple primary lung cancer (MPLC) refers to the simultaneous occurrence of two or more lung primary malignant tumors in one individual. The detection rate of MPLC has increased significantly in recent years, and the distinction between MPLC and lung metastasis has strong clinical significance. Whole exome sequencing (WES) can clearly identify the heterogeneity between MPLC nodules. Here, we report a case of a 50‐year‐old Asian female without a history of smoking. She underwent a lung computed tomography (CT) scan and three ground‐glass nodules (GGNs) were found which were pathologically confirmed as atypical adenomatous hyperplasia (AAH), minimally invasive adenocarcinoma (MIA) and invasive adenocarcinoma (IA), respectively. We performed WES on the three pulmonary nodules and analyzed the sequencing results. We believe that this is the first published report of a case of “three phases” of lung adenocarcinoma analyzed by WES. Under the same genetic background and internal environment, these three nodules showed significant genetic differences and developed into “three phases” of lung adenocarcinoma. Analysis of the WES results supported the lung adenocarcinoma model from AAH to MIA and IA, and explored possible potential driver genes and therapeutic targets.

**Key points:**

**Significant findings of the study:**

We used WES to analyze the gene mutation status of three tumors in one individual. We found that even if under the same genetic background, AAH, MIA and IA showed significant genetic differences and developed into “three phases” of lung adenocarcinoma.

**What this study adds:**

Analysis of the WES results supported the lung adenocarcinoma model from AAH to MIA and IA, and explored possible potential driver genes and therapeutic targets.

## Graphical abstract

We analyzed the origin and evolution of lung adenocarcinoma based on the WES results of MPLC in one individual. Analysis of the WES results reflects a progressive genomic evolution at the single nucleotide level, which we believe supports the lung adenocarcinoma model from AAH to MIA and ADC. Meanwhile, we explored possible potential driver genes and therapeutic targets.

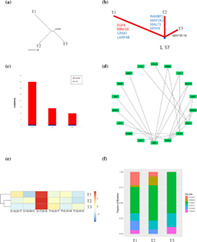

## Introduction

Multiple primary lung cancer (MPLC) refers to the simultaneous occurrence of two or more lung primary malignant tumors in the same or different lobes of the lungs in one individual.^1^ In the past, MPLC was considered a rare disease, with the incidence of MPLC reported to be 0.2%–8%.^2^ However, the incidence of MPLC is increasing with the popularity of low‐dose spiral computed tomography (CT) in recent years, and more cases of MPLC are being diagnosed.^3^ Determining whether the tumor is primary or metastatic has been a longstanding clinical dilemma. Clarifying the relationship between multiple pulmonary nodules can help to determine the tumor stage, make treatment decisions and assess the prognosis of patients.^4^ Whole exome sequencing (WES) can help us to understand and distinguish each MPLC lesion genetically, which is very useful for determining the origin and clonal evolution of nodules. In this study, we report a special case of triple synchronous primary lung cancer, consisting of three types: atypical adenomatous hyperplasia (AAH), minimally invasive adenocarcinoma (MIA), and invasive adenocarcinoma (IA). These three pathological types are considered as the “three phases” in the evolution of lung adenocarcinoma.^5^ However, the molecular pattern and evolutionary trajectory from AAH to IA remains controversial.^6^ After obtaining patient approval, we performed WES on these three tumors. High genetic heterogeneity suggested that the three nodules were independent of each other. We analyzed the gene sequencing results to identify the major genes that may play an important role in the “three phases” of lung adenocarcinoma.

## Case report

**Figure 1:**
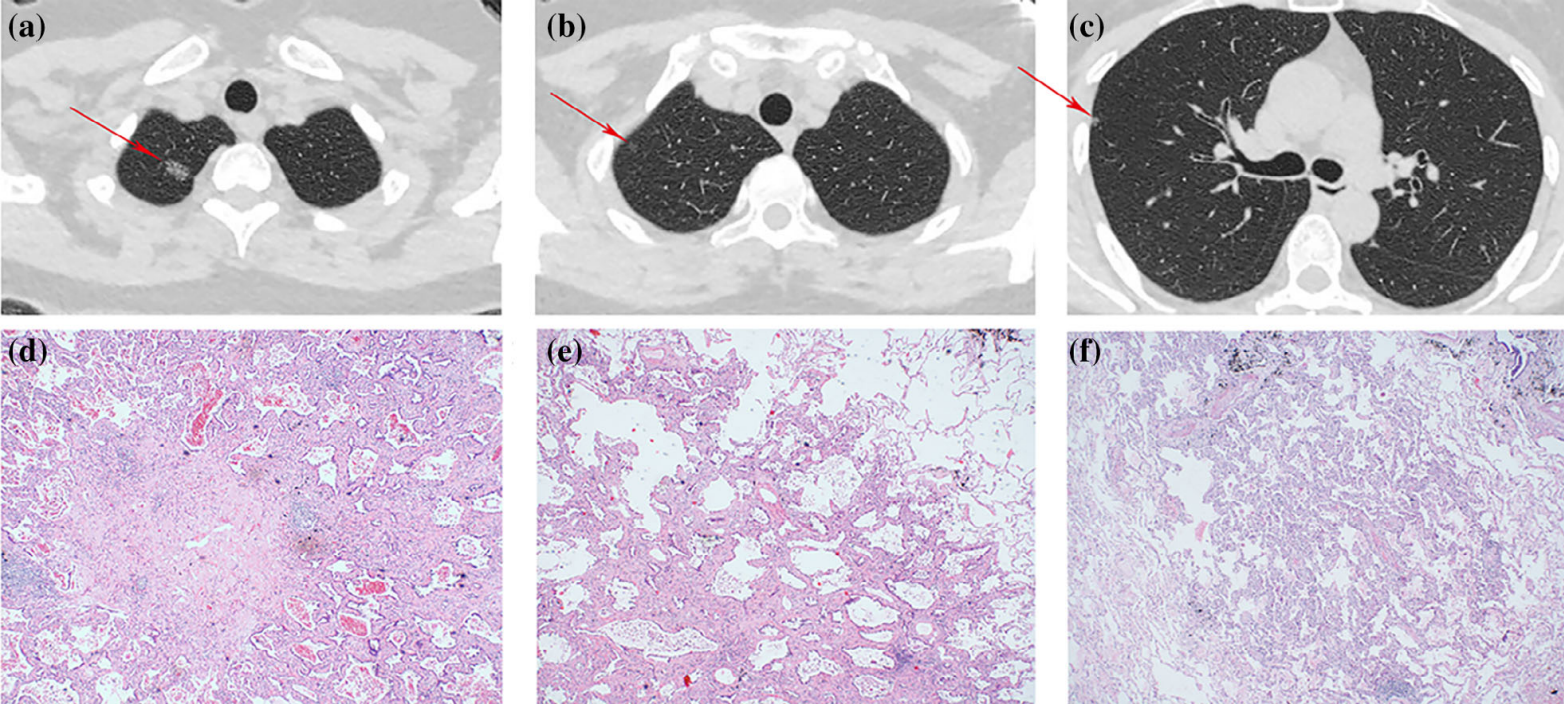
(**a**, **b**, **c**) Lung computed tomography (CT) scan. The patient's preoperative lung CT scan is shown; the red arrow indicates the tumors located in the right upper lobe of the lung (**a**, **b**, and **c** refer to IA, MIA and AAH, respectively). (**d**, **e**, **f**) Pathological images (**d**, **e**, and **f** refer to IA, MIA and AAH, respectively).

A 50‐year‐old Asian female patient with no history of smoking was found to have three ground‐glass nodules (GGNs) in the right upper lobe of her lung in March 2017 (Fig 1a–c). She was subsequently admitted to hospital for right upper lobe lobectomy and lymphadenectomy. Three nodules were found in the resected lobe which were pathologically diagnosed as AAH (Fig 1f), MIA (Fig 1e), and IA (Fig 1d). AAH was located in the S2 bronchopulmonary segment, and was clinically staged as cT1N0M0. The pathological stage was pT1N0. MIA was located in the S1 bronchopulmonary segment, and was clinically staged as cT1N0M0. The pathological stage was pT1N0. IA was also located in S1 bronchopulmonary segment, and was clinically staged as cT1N0M0. The pathological stage was pT1N0. Both MIA and IA have an adherent growth pattern with a gradual transition from the surrounding alveolar epithelium, and pathologists considered these two nodules to be primary tumors rather than intrapulmonary metastases. To clarify the relationship and differences between these three tumors at the genetic level, we performed WES on three pulmonary nodules using Illumina NovaSeq sequencer, and analyzed and compared the sequencing results of different nodules.

**Figure 2:**
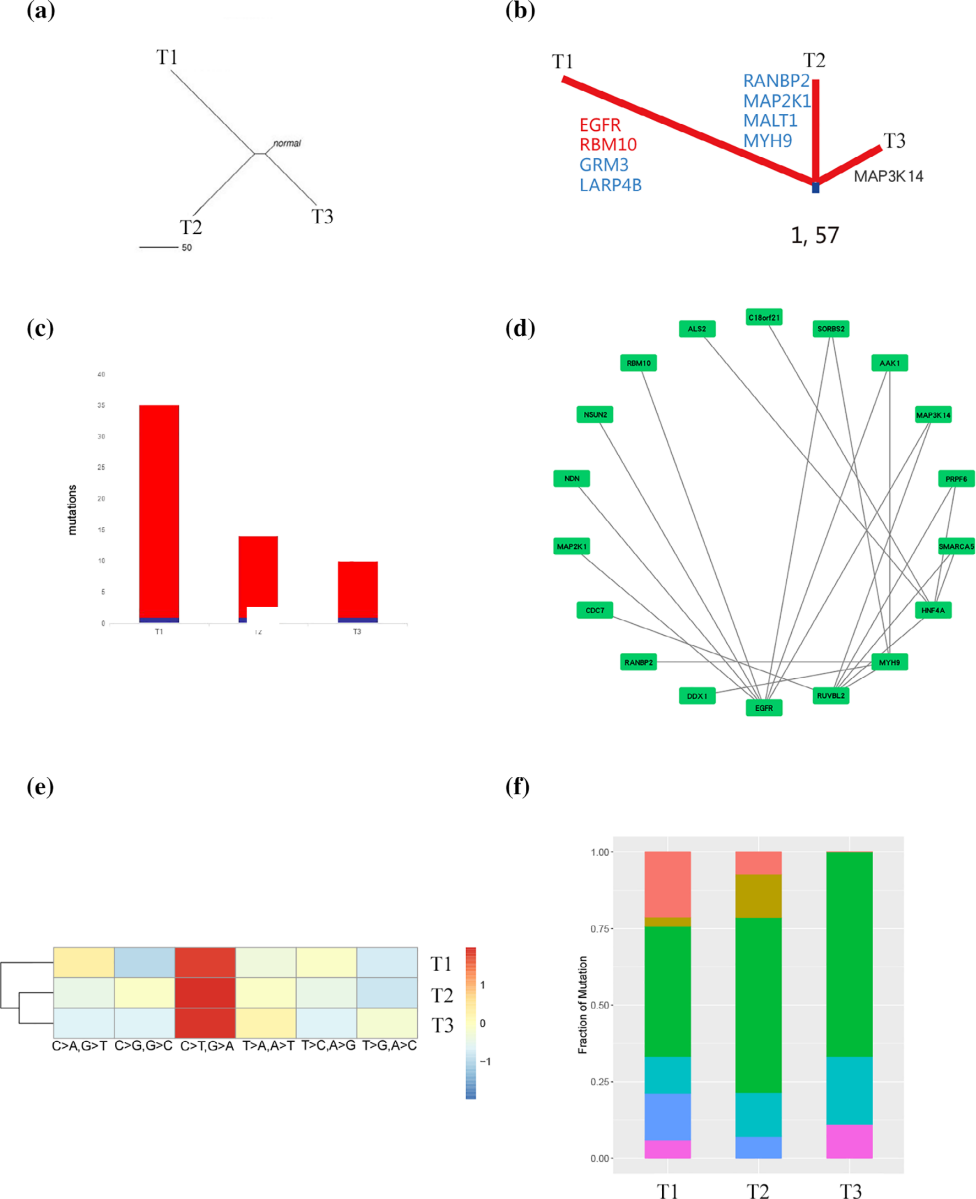
T1, invasive adenocarcinoma; T2, minimally invasive adenocarcinoma; T3, atypical adenomatous hyperplasia. (**a**) Dispersion analysis. This shows the dispersion analysis of the three tumors in this patient. (**b**) Phylogenetic tree. The numbers represent the truncal mutational burden and total mutational burden for all sectors of IA, MIA and AAH. The total mutational burden for all sectors is the sum of the mutations in each lesion of the patient. The three lines represent the number of mutations in the IA, MIA and AAH lesions. The highly expressed genes in IA, MIA and AAH are listed and the specific genes recorded in the COSMIC Cancer Gene Census are in blue and the LUAD‐specific driver genes are in red. (**c**) Comparison of the tumor mutational burden in different lesions. According to the somatic cell mutation data, we counted the number of nonsynonymous mutations out of all the somatic cell mutations to obtain the tumor mutational burden of each tumor type. (**d**) Protein‐protein interaction (PPI) network. AAH, MIA, and IA had different gene mutation status. *EGFR* L858R mutation was only found in IA, not in other earlier phase nodules. In order to observe the interaction of the mutant genes and whether a mutant gene could affect major driver genes, the protein‐protein interactions (PPIs) were retrieved from the InWeb_InBioMap database, and Cytoscape was used to visualize the network in order to find the differences and relationships among these three phase nodules. (**e**) Mutation spectrum clustering heat map. This map shows a mutation spectrum, which expresses the different proportions of different types of mutations in IA, MIA and AAH using different colors. The abscissa shows the six mutation types, and the ordinate are the different tumors; the redder the heat map grid, the greater the proportion of this type of mutation in the corresponding tumor, while the bluer the grid, the smaller the proportion of this type of mutation in the corresponding tumor. (**f**) Mutation spectrum statistics. The abscissa represents the different tumors, while the ordinate shows the ratio of different types of mutant bases. This graph illustrates the different proportions of different types of mutant bases. (**c**) (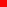) Non‐trunk; (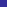) Trunk; (**f**) Mut_type: (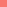) C>A, G>T, (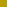) C>G, G>C, (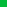) C>T, G>A, (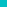) T>A, A>T, (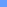) T>C, A>G, (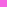) T>G, A>C.

Under the same genetic background and internal environment, three different pathologic and molecular types of tumors coexisted in the lung of this patient. Dispersion analysis enabled us to better identify the evolutionary relationship between the three tumors. We subsequently plotted a dispersion analysis diagram of the three nodules in this patient, which showed that the dispersion between the three nodules was 50% (Fig 2a). This means that there are similarities and differences between AAH, MIA and IA.

Using the presence and absence of somatic mutations in tumors, we first calculated the genetic distances between tumors using the Hamming distance. Then we transformed the distance into a tree by the Neighbor‐Joining and UPGMA tree drawing method from PHYLIP 3.6.^7^ We mapped a phylogenetic tree of the three tumors in this patient to reconstruct their genetic evolution pattern (Fig 2b). The trunk represents the initial events of cancer, while the branches represent the subsequent events acquired during carcinogenesis. The three tumors had a total of 57 gene mutations and a common gene mutation, BAGE2. IA contains two LUAD‐specific driver genes and two driver genes included in the Cancer Gene Census (CGC) in the Catalogue of Somatic Mutations in Cancer (COSMIC). There are four driver genes included in the CGC in COSMIC in MIA, and AAH does not have a driver gene.

We used MuTect2 (GATK V3.6) software to analyze somatic mutations. Credible somatic mutation data were then screened out according to the mutation depth and other information, and the number of nonsynonymous mutations in the credible somatic mutation was counted to obtain the tumor mutational burden (TMB) of the tumor tissues. AAH showed 10 mutation sites, MIA showed 14 mutation sites, and IA showed 35 mutation sites (Fig 2c). The TMB of the three tumors was relatively low, especially in AAH, and the TMB of AAH, and MIA and IA increased successively.

In order to observe the interaction of the mutant genes and whether a mutant gene could affect major driver genes, the protein‐protein interactions (PPIs) were retrieved from the InWeb_InBioMap database,^8^ and Cytoscape^9^ was used to visualize the network. We found that *EGFR* L858R mutation was observed only in IA and had the most interactions with other mutant genes (Fig 2d). In addition, we observed two other important genes, MAP3K14 and MAP2K1. MAP3K14 mutation is observed in AAH, while MAP2K1 mutation appears in MIA. Both MAP3K14 and MAP2K1 can interact with EGFR, and MAP3K14, MAP2K1 and EGFR are important components of MAPK signaling pathway.

We used Unified Genotyper, Haplotype Caller and atlas2 software to analyze genetic single nucleotide polymorphism (SNPs). Atlas and platypus software were used to analyze insert deletion mutations (Indel) of heritability. Annovar software was used to perform functional annotation for detected gene mutations and complete the detection of inherited gene mutations. The proportion of the six single bases was calculated based on the single‐nucleotide variant (SNV) results of the three tumors: C>A/G>T, C>G/G>C, C>T/G>A, T>A/A>T, T>C/A>G, T>G/A>C. This directly reflects the preference of the mutation type of each tumor sample. All three tumors showed dominant substitution of C > T/G > A (Fig 2e,f). In AAH, MIA and IA, the proportion of C > T/G > A substitution decrease in order. However, the substitution of C>A/G>T and T>C/A>G was very rare in the AAH. In AAH, MIA and IA, the proportion of C>A/G>T substitutions is increasing in turn, as well as T>C/A>G substitutions.

## Discussion

In this report, we describe a rare case of AAH, MIA and IA coexisting in one individual. We performed WES on the three tumors and analyzed their clonal relationships. We found that under the same germline characteristics and environmental exposure factors, the three tumors presented different phenotypes and developed into “three phases” of lung adenocarcinoma.

We observed a 50% dispersion among the three tumors (Fig 2a), suggesting that the three tumors were independent with some degree of similarity. *BAGE2* mutation was the only common mutation among the three tumors, and the abundance of *BAGE2* mutation increased successively in AAH, MIA, and IA (Fig 2b). *BAGE* genes were not included in COSMIC. However, previous studies have confirmed that *BAGE* genes are expressed in melanoma, bladder cancer, lung cancer and some other histological types of tumors.^10^ *BAGE* are candidate genes encoding tumor antigens, and the antigens encoded by them may prove useful in tumor immunotherapy.^10^, ^11^ However, no studies have so far been published on the biological mechanism of *BAGE2* in lung adenocarcinoma. We believe that *BAGE2* mutation appears at the AAH stage, and its mutation abundance increases gradually during the development of lung adenocarcinoma. The *BAGE2* gene may be further explored as an important site for immunotherapy for lung adenocarcinoma in the future.

TMB refers to the total number of somatic mutations per million bases in the coding region of tumor cells.^12^ It is a biomarker that reflects the level of tumor mutation.^13^ We observed low TMB in all three tumors (Fig 2c). TMB can be affected by factors such as age and tobacco, and the TMB of adenocarcinoma is relatively low. However, the patient in this study was a relatively young non‐smoker pathologically diagnosed with adenocarcinoma which we believed to be the reason for the low TMB of the three tumors. Neoantigens in tumors are associated with TMB, and patients with higher TMB are more likely to produce immune‐stimulating neoantigens, and were of the opinion that these three tumors would have limited benefit from immunotherapy. In addition, TMB has been reported to be affected by C>A/G>T substitution.^14^ We observed a gradual increase of C>A/G>T substitution in AAH, MIA and IA, as well as TMB. This is consistent with previous studies.^14^ During the development of AAH to IA, the TMB gradually increased, indicating that the mutation frequency increased during the evolution of adenocarcinoma. This also suggests that a progressive accumulation of SNVs is accompanied by the development of early tumors.

AAH, MIA, and IA are three‐phase nodules for lung adenocarcinoma. In the case reported here, *EGFR* L858R mutation was only observed in IA, and not in AAH and MIA. We propose that *EGFR* mutation occurred in the development of adenocarcinoma. As a driver gene, *EGFR* may promote adenocarcinoma progression. In comparison, AAH and MIA progressed very slowly. More and more MPLCs are found nowadays, and surgery has been regarded as the main therapy for this kind of patient. No standard postoperative therapies have as yet been suggested. However, sometimes surgery is not the best solution, especially where there are multiple nodules present. From this case, we found target therapy for multiple nodules with IA was a good choice, but not suitable for nodules with early phases of adenocarcinoma such as AAH and MIA. However, more clinical samples are needed to verify this.

Furthermore, in the process of gene interaction analysis, we identified three important genes that may play an important role in the evolution of lung adenocarcinoma (Fig 2d). These three genes were *MAP3K14*, *MAP2K1* and *EGFR*, found in AAH, MIA and IA, respectively. They are all significant coding genes in the MAPK signaling pathway. *EGFR* binds to epidermal growth factor and activates the classical MAP kinase pathway. MEK1, encoded by *MAP2K1*, acts as a MAP kinase (MAPKK), and can stimulate the enzymatic activity of ERK (MAPK). Thus, it can be seen that in the classical MAP kinase pathway, MAP2K1 is located in the downstream of EGFR. The third gene, *MAP3K14*, found in AAH, is defined as MAPKKK and is located at the most downstream of the MAPK signaling pathway.^15^, ^16^, ^17^ This reflects a progressive genomic evolution at the single nucleotide level, which we believe supports the lung adenocarcinoma model from AAH to MIA and IA. MAP3K14 and MAP2K1 may serve as potential therapeutic targets in the treatment of lung adenocarcinoma in the future.

These three tumors shared the same genetic background and exposed environment, so the mutant process in the context of independent tumors was explored. According to the proportion of six single bases replacement types, we plotted the mutation spectrum (Fig 2f) of base replacement and the clustering heat map of mutation spectrum (Fig 2e). Previous studies have shown that there is a difference in the mutation spectrum between smokers and non‐smokers. C>A/G>T transversions were the most common type among smokers and C>T/G>A transversions were the most frequent type among non‐smokers.^18^ This patient had no history of smoking, and the mutation spectrum showed C>T/G>A transversions as the dominant mutation, which is consistent with previous reports. In most human cancer types, C>T/G>A transversions have been reported to show a strong positive correlation with age.^19^ C>T/G>A transitions mainly occurs at NpCpG trinucleotides which is caused by the relative increase rate of 5‐methylcytosine spontaneous deamination. They operate in germline and normal somatic cells, resulting in substantial depletion of NpCpG sequences.^19^ The patient in our report was 50 years old with a major conversion type consistent with that described in previous studies. However, during the evolution from AAH to IA, the proportion of C > T/G > A gradually decreased, while the proportion of C>A/G>T and T>C/A>G increased. We believe that the reason for this is that most C>T/G>A transversions occur continuously and steadily, while other mutations increase gradually after exposure to different carcinogens during the development and progression of adenocarcinoma.

In conclusion, we analyzed the origin and evolution of lung adenocarcinoma based on the WES results of MPLC in one individual. Analysis of AAH, MIA, and IA under the same germline characteristics and exposure factors revealed evidence of convergent evolution. However, more case samples are needed to validate the findings in this report. The carcinogenesis model of early lung adenocarcinoma, the identification of more driver genes and therapeutic targets should be explored via further molecular biological studies.

## Disclosure

The authors declare that they have no conflict of interest.
